# Adsorption behavior of tin phthalocyanine onto the (110) face of rutile TiO_2_

**DOI:** 10.3762/bjnano.11.67

**Published:** 2020-05-26

**Authors:** Lukasz Bodek, Mads Engelund, Aleksandra Cebrat, Bartosz Such

**Affiliations:** 1 Faculty of Physics, Astronomy and Applied Computer Science, Jagiellonian University, ul. S. Lojasiewicza 11, 30-348 Krakow, Poland; 2 Espeem S.A.R.L., c/o Technoport S.A., 9 Avenue des Haut-Fourneaux, L-4362 Esch-Sur-Alzette, Luxembourg; 3 EMPA, Swiss Federal Laboratories for Materials Science and Technology, Überlandstrasse 129, 8600 Dübendorf, Switzerland

**Keywords:** rutile (110) surface, scanning tunneling microscopy (STM), tin phthalocyanine (SnPc), titanium dioxide (TiO_2_)

## Abstract

The adsorption behavior of tin phthalocyanine (SnPc) molecules on rutile TiO_2_(110) was studied by scanning tunneling microscopy (STM). Low-temperature STM measurements of single molecules reveal the coexistence of two conformations of molecules on the TiO_2_ surface. Density functional theory-based simulations (DFT) indicate that the difference originates from the position of the tin atom protruding from the molecule plane. The irreversible switching of Sn-up molecules into the Sn-down conformation was observed either after sample annealing at 200 °C or as a result of tip-induced manipulation. Room-temperature measurements conducted for a coverage of close to a monolayer showed no tendency for molecular arrangement.

## Introduction

Phthalocyanines (Pcs) are aromatic molecules that can form metal complexes with a variety of elements, which can be used to tune molecular properties, such as position or shape of adsorption bands. Therefore, Pcs show tremendous potential for a multitude of applications [1–5]. In many applications, the interface between a Pc molecule and the surface onto which it is adsorbed is of paramount importance. It is the adsorption configuration that affects phenomena such as charge transfer and layer stability. Since metal oxides are commonly used as support for the growth of molecular layers in many technological solutions, it is not surprising that Pcs on titanium dioxide faces have been widely studied. Most studies of phthalocyanine adsorption on rutile (110) and (011) faces consider flat Pcs (CoPc [6], CuPc [7–10], ZnPc [11], FePc [12] and H_2_Pc [13]), while their nonplanar counterparts such as SnPc have been rarely probed [14]. Spectroscopy studies or density functional theory (DFT) calculations of molecular species adsorbed onto titanium dioxide can be an arduous task, especially for big molecules such as phthalocyanines. However, studies of either the morphology of assemblies or the behavior of isolated molecules by microscopic methods may shed light on the role of the central atom of Pcs in the adsorption process and allow one to distinguish it from the influence of the surface orientation. To fill this gap in knowledge, here, we report room-temperature (RT) and low-temperature (LT) STM-based studies of the adsorption of nonplanar tin phthalocyanine (SnPc) molecules a rutile (110)-1 × 1 surface of TiO_2_. SnPc molecules appear on such a surface in two flat-lying geometries defined by the position of a tin atom protruding from the macrocycle: “Sn-up” and “Sn-down”. Switching from the Sn-up to the Sn-down geometry can be realized by annealing the sample at 200 °C or by tip-induced manipulation (bias pulse). X-ray photoelectron spectroscopy (XPS) measurements reveal a lack of strong interactions between SnPc molecules and the rutile surface, while DFT-based simulations combined with high-resolution STM images imply that a Sn-down geometry may be preferred as a consequence of steric adjustment between the molecular shape and the corrugated (110) surface.

## Experimental

The experiment was carried out in two ultrahigh vacuum (UHV) systems with a base pressure below 2 × 10^−10^ mbar. A rutile TiO_2_(110) crystal (produced by MaTeck GmbH) was mounted onto a sample holder allowing for the direct heating by using an AC electric current. An atomically flat and clean surface (as checked by STM) was prepared by repetitive cycles of Ar^+^-ion bombardment at an energy of 1 keV and subsequent annealing to a temperature of 700 °C. Tin phthalocyanine molecules (Tokyo Chemical Industry Co., Ltd.) were thermally evaporated by using an effusion cell (Kentax GmbH). After prudent degassing, the deposition flux rate (0.25 ML/min) was determined using a quartz crystal microbalance. Scanning tunneling microscopy (STM) experiments were performed with the use of either a low-temperature STM (LT-STM) operating at ca. 78 K or a room-temperature STM (RT-STM) manufactured by Scienta Omicron installed in a separate UHV system. Electrochemically etched Pt–Ir tips were used as probes. Stable empty-state STM images were collected in a constant-current mode (tunneling current *I*_t_ < 15 pA; bias voltage *U*_tip_ < 2 V). X-ray photoelectron spectroscopy (XPS) was carried out by using a VG Scientific X-ray source Mark II (Mg Kα) combined with a Scienta EAC2200 Nanosam 570 analyzer. Ti 2p peaks were used for global calibration of the XPS spectra. A Shirley function was used for background correction during XPS data analysis. Calculations were performed by employing density-functional theory (DFT) using the SIESTA code [15] with a double-zeta polarized (DZP) basis set and orbital radii defined using an energy shift of 100 meV, a Perdew–Burke–Ernzerhof (PBE) exchange–correlation potential [16] and a real-space grid equivalent to a plane-wave cutoff of 200 Ry. Forces were relaxed until they were smaller than 0.020 eV·Å^−1^. To compute the STM images, the surface integration technique of Paz and Soler was followed [17] with the Tersoff–Hamann approximation [18], assuming a proportionality factor of 1.00 nA·Å^−3^ for the ratio between the local density of states and the current. To mimic the effect of spatial uncertainty in the measurement, which reduces the resolution of the STM images, currents were convoluted with a Gaussian kernel,


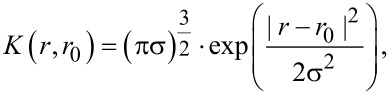


with σ = 0.7 Å. Similarly, a spatial broadening of 0.01 and 1.20 Å was introduced for the identification of the impact of the broadening.

## Results and Discussion

**Figure 1:**
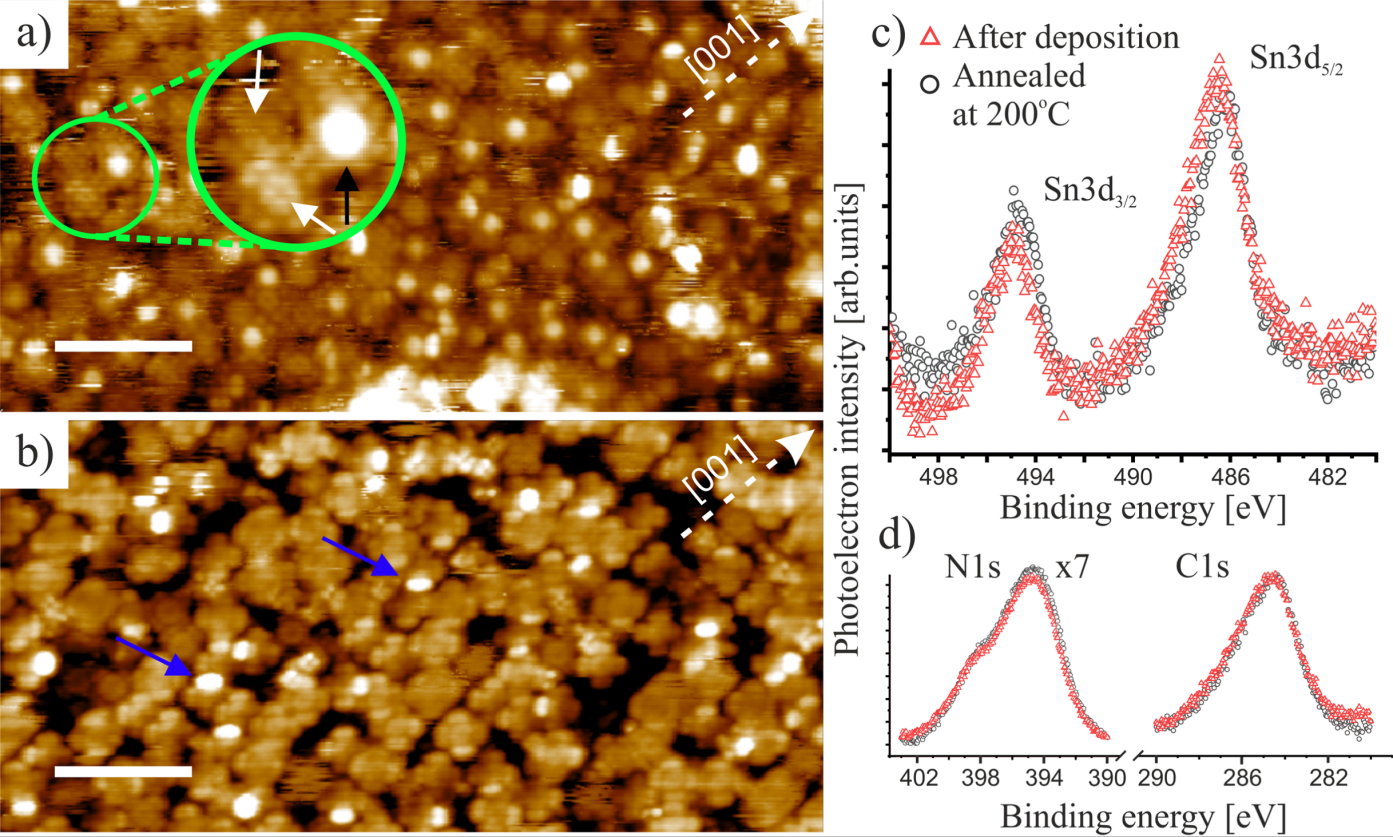
a) Empty-state RT-STM image of a SnPc monolayer directly after deposition with both geometries of the molecules present; in the inset marked by the green circle, an area containing three molecules is presented. Two of the molecules do not have a central protrusion (marked by white arrows) and one of the molecules has a central protrusion (marked by the black arrow); b) empty-state RT-STM image of a SnPc monolayer after annealing at 200 °C; c) XPS spectra of the Sn 3d core level; d) XPS N 1s and C 1s core-level peaks measured for a SnPc monolayer before and after sample annealing at 200 °C, which do not show any significant difference. Scanning parameters: 50 × 50 nm^2^; a) *I*_t_ = 1.3 pA, b) *I*_t_ = 3.3 pA; *U*_tip_ = 1 V. Scale bars: 5 nm.

Isolated SnPc molecules are highly mobile on TiO_2_(110) at room temperature. Their mobility is revealed on STM maps by the observation of streaky lines and disappearing shapes (Figure S1, Supporting Information File 1). The features are blurred even when probed using currents with a magnitude of a few picoamperes. With increasing coverage, a few stable single molecules can be found, which are most likely stabilized by surface defects, as well as stable molecular assemblies in limited areas close to terrace edges, where movement is hampered by steric interactions (Figure S2, Supporting Information File 1). The intramolecular interactions lead to the stabilization of the layer at a coverage of close to 1 ML. The STM image of a monolayer of SnPc taken directly after deposition is presented in Figure 1a. Neither short- nor long-range order is observed. The adsorbed molecules are flat with the macrocycle lying parallel to the surface. Additionally, tin phthalocyanine molecules on the surface adopt two distinct geometries and can be observed with either a protrusion in the center of the macrocycle (see the black arrow inside the green circle in the Figure 1a) or as molecules devoid of such a feature (see the white arrows inside the green circle in Figure 1a). Molecules with a bright spot amount to approximately (30 ± 7)% of all observed molecules, regardless of the sample temperature during deposition (up to 100 °C). Moreover, this ratio does not change noticeably for samples measured after being left in UHV for a few days. However, after post-deposition sample annealing at 200 °C, only molecules without a central protrusion are present on the surface (Figure 1b), while the total amount of adsorbed molecules remains the same as before the thermal treatment. Other noticeable bright points originate from overlapping pyrrole-like subunits of adjacent phthalocyanines, as indicated by the blue arrows in Figure 1b. The layer is stable up to approximately 280 °C, when the molecules start to desorb from the surface. No changes in the layer morphology (i.e., molecular organization) were observed upon annealing. Both types of SnPc molecules observed have the same contour of the macrocycle (see the green circle in Figure 1a), suggesting that in both geometries the molecules remain flat on the surface.

To determine the nature of the molecule–substrate bond and its change due to the switch in the molecular geometry, we performed XPS measurements. Signals corresponding to the Sn 3d, N 1s and C 1s lines were collected from a freshly formed sample with molecules in both geometries present as well as from a sample annealed at 200 °C. As shown in Figure 1c and Figure 1d, neither shifts nor new lines were detected suggesting that the switching of the molecular geometry is not accompanied by the formation of a new chemical bond between the molecule and the substrate. Additionally, the XPS results allow one to exclude demetalation [19] as a possible explanation for the observed changes. In a SnPc molecule, a tin atom protrudes from the macrocycle making the molecule nonplanar [20]. This enables the molecule to adsorb onto a surface with a tin atom pointing either towards the surface (Sn-down) or towards the vacuum (Sn-up). Similar findings were reported for the Ag(111) surface [21–22], where Sn-up Pc molecules appeared with bright central protrusions, while Sn-down Pc molecules exhibited a depression in the center of the macrocycle.

**Figure 2:**
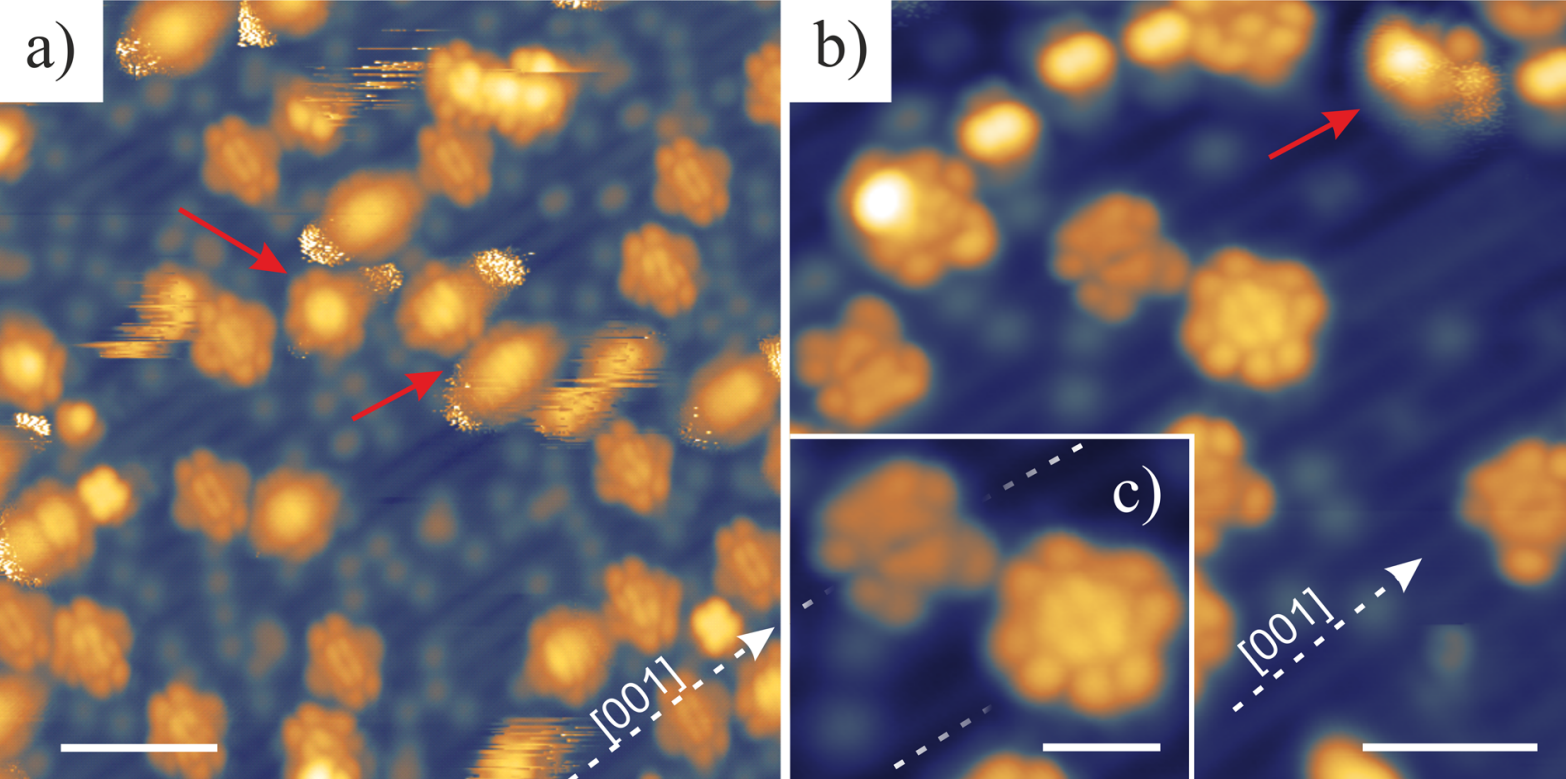
a, b) Set of high-resolution LT-STM images presenting SnPc molecules adsorbed onto a rutile (110) surface with c) inset image showing the varying molecular arrangement of Sn-down molecules with respect to the [1] direction of the surface oxygen rows marked by a white dashed line. Molecules without protrusion (Sn-down) in the center remain stable, whereas some tip-induced movement can be observed for Sn-up Pc molecules, as indicated by the red arrows. Scanning parameters: a) 15 × 15 nm^2^, b) 9 × 9 nm^2^, c) 3.4 × 2.9 nm^2^; *I*_t_ = 15 pA; *U*_tip_ = 1.6 V. Scale bars: a) 3 nm, b) 2 nm and c) 1 nm.

To reduce the mobility of isolated molecules and to precisely determine their adsorption positions, STM measurements at low coverage (approx. 0.3 ML) were carried out under cryogenic conditions (78 K) as presented in Figure 2. Both types of SnPc molecules (with and without central protrusion) appear on the surface, as was observed for the non-annealed monolayer at room temperature (Figure 1a). Between the molecules, oxygen rows can be recognized (imaged as stripes in the [001] direction), which are characteristic for a clean surface [23]. As shown in Figure 2, single point protrusions located on the dark rows mostly correspond to hydroxy groups [24]. Sn-down Pcs may adopt two different positions, as shown in Figure 2b. The inset image (Figure 2c) shows that the left molecule lies above an oxygen row with pyrrole-like subunits located near adjacent titanium rows, while the right Sn-down molecule is adsorbed with its diagonal aligned parallel to the [001] direction on the titanium rows. Sn-up molecules also adsorb over the STM bright surface rows. Approximately 90% of observed SnPc molecules are adsorbed with the long axis along the oxygen rows. It is noteworthy that Sn-down molecules remain stable under the scanning conditions (LT), while the interaction with the tip induces movement of Sn-up Pcs, as indicated by the red arrows in Figure 2.

Additionally, some of the Sn-down Pc molecules are probed as asymmetric or elongated. A similar effect was extensively studied in the case of negatively charged CuPc adsorbed onto NaCl/Cu(100) [25], when controlled changes in the position of the molecular orbitals by manipulating adatoms in the vicinity of the molecule were presented. We assume that the shape asymmetry of SnPc may also appear as a result of the presence of the surrounding molecules, or it can arise from the corrugated nature of the rutile (110) surface including its defects.

**Figure 3:**
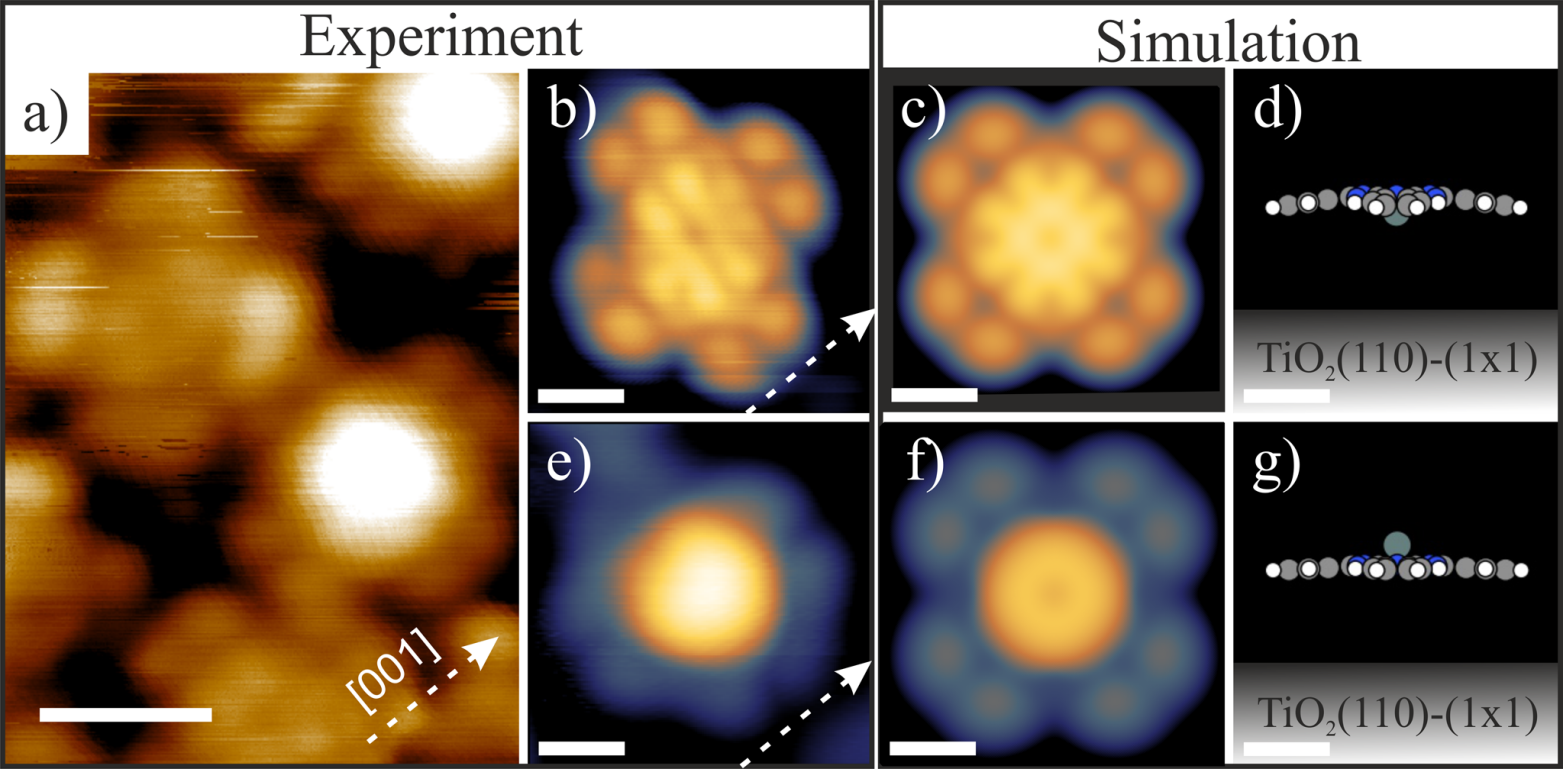
a) RT-STM image of SnPc molecules in both geometries; b) LT-STM image of a molecule identified as Sn-down Pc; c) empty-state STM image simulation obtained for the lowest unoccupied molecular orbital (LUMO) calculated for d) a DFT-optimized geometry for a Sn-down molecule influenced by the surface; e) LT-STM image of a Sn-up molecule with f) the corresponding simulated STM image at the LUMO energy level and g) optimized DFT gas-phase geometry. Scanning parameters: a) 3 × 4 nm^2^, *I*_t_ = 3 pA, *U*_tip_ = 1.6 V; b) 2 × 2 nm^2^; *I*_t_ = 1.5 pA; *U*_tip_ = 2 V; e) 2 × 2 nm^2^, *I*_t_ = 10 pA, *U*_tip_ = 1.6 V. Scale bars: a) 1 nm and b–g) 0.5 nm.

To confirm the identification of Sn-up and Sn-down geometries, we performed DFT calculations. A comparison of experimental and simulated images of SnPc molecules is presented in Figure 3.

To discuss the molecular geometry on the surface, we combined RT-STM images of SnPc molecules forming a monolayer (Figure 3a) with high-resolution LT-STM images of isolated molecules. Experimental data are compared with simulated STM images at the LUMO energy level of molecules in a DFT-optimized geometry. Figure 3b presents an LT-STM image of an isolated Sn-down molecule with the corresponding simulated image presented in Figure 3c. A side view of the DFT-optimized geometry for the Sn-down Pc molecule is presented in Figure 3d. An analogous combination of LT-STM image, DFT-simulated image and optimized geometry for a Sn-up molecule is presented in Figure 3e–g.

A DFT-simulated image of a Sn-up molecule in the gas-phase geometry corresponds well to the experimental results. The bright protrusion centered around the position of the metal atom dominates the image. At the periphery of the molecule, four weaker double protrusions are visible that are located over the pyrrole-like subunits of Pc. This close resemblance for both images suggests that a SnPc molecule with the metal atom pointing up is not strongly deformed on the surface.

In contrast, simulated images of gas-phase SnPc in the Sn-down position do not recreate the experimental results (marked by the blue square in Figure S3, Supporting Information File 1). Especially, the pyrrole-like subunits appear much brighter than observed in the experimental data. Therefore, molecules in the Sn-down geometry cannot be modeled while disregarding the presence of the substrate. Hence, we introduced a simple heuristic model for the interaction between the molecule and the surface (see Calculation Methodology in Supporting Information File 1). The interaction leads to a bending of the pyrrole-like subunits of the molecule towards the surface. Simulated STM images of a molecule relaxed in this way are in very good agreement with the experimental images. The Sn-down Pc molecule appears as a flower-like shape with four bright arms. In addition, the structure in the center of the molecule is well reproduced in the simulated image including the relative intensities of different parts of the molecule.

**Figure 4:**
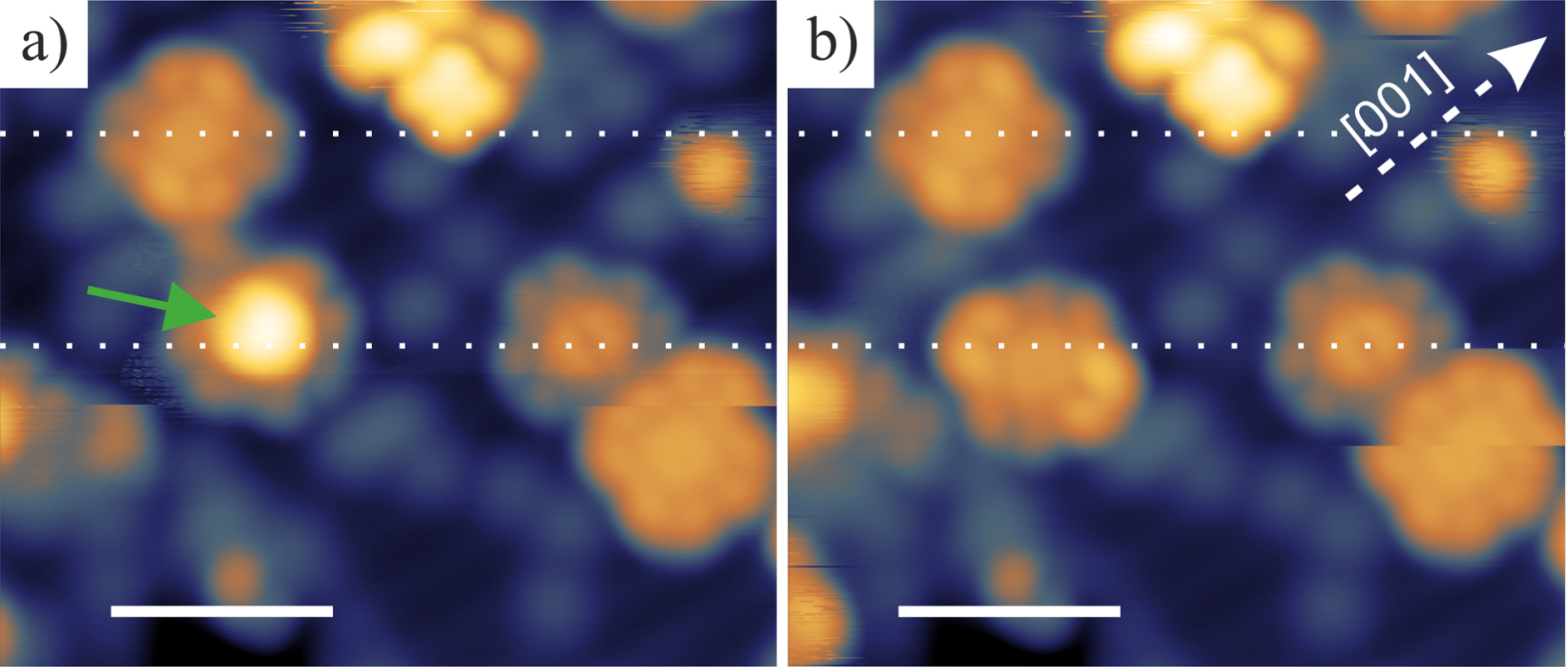
a) LT-STM image before application of a pulse voltage (3 V) above a Sn-up Pc molecule protrusion (indicated by the green arrow) and b) image of the same region taken after the manipulation. As a result, a switching of the molecule from Sn-up to Sn-down occurs. The horizontal white dotted lines show the position of intact molecules. Scanning parameters: 7 × 6 nm^2^; *I*_t_ = 10 pA; *U*_tip_ = 1.6 V. Scale bars: 2 nm.

The change in orientation of SnPc molecules on the TiO_2_(110) surface from Sn-up to Sn-down described in the previous paragraph is induced by thermal energy. However, it is also possible to induce irreversible switching of the molecular geometry by application of a voltage pulse applied from the STM tip. Figure 4 presents the area before (Figure 4a) and after (Figure 4b) application of a bias voltage pulse above the center of a Sn-up Pc molecule, pointed out by the green arrow. White dashed lines indicate the position of intact molecules. A bias voltage of +3 V (*t* < 50 ms) was found to be sufficient to induce switching. A bias pulse converts a Sn-up molecule into a Sn-down molecule, but additional effects such as molecule movement and rotation of the macrocycle are typically observed. Additionally, only switching from Sn-up to Sn-down is observed during the experiment. The opposite process (Sn-down to Sn-up) does not occur. A Sn-down Pc molecule subjected to similar voltage pulse parameters remains unchanged. Application of pulses with higher magnitudes (above 5 V) leads to a lateral movement of the molecules or to their distortion.

The conformational switching of SnPc molecules was previously discussed for the Ag(111) surface [21], the InAs(111) surface [26] and a 1 ML PTCDA/Ag(111) interface [27]. Using a C_60_-functionalized tip, successful switching between Sn-up and Sn-down was also reported on Cu(111) and Ag(100), while this reaction did not occur on Au(111) and Au(110) surfaces [28]. On the Ag(111) surface, within the first layer, Sn-up molecules were irreversibly switched to the down position, while on InAs(111), the molecules could be switched freely between both geometries. On Ag(111), the free switching appeared in subsequent layers. This mainly suggests that the surface influences the switching process and indicates that the Sn-down Pc position is preferable. Here, tip-induced irreversible switching was observed on a rutile (110) surface. In our previous work [14], the adsorption process of SnPc on the (011) face of rutile TiO_2_ was studied by microscopic methods. Up to a monolayer, SnPc molecules exhibit comparable behavior on both rutile surfaces including the observation of a very short residence time under the tip apex at room temperature. The difference in the adsorption process between (110) and (011) surfaces is clear when increasingly more molecules are evaporated onto the surface. After deposition of the equivalent of a few monolayers, the formation of well-ordered phases of non-flat lying molecules is observed on the rutile (011) surface. A similar amount of molecules deposited onto the rutile (110) surface does not lead to the formation of such phases, and, instead, layer-by-layer deposition without any particular arrangement is observed (Figure S3, Supporting Information File 1). This outcome may suggest that for SnPc/TiO_2_(011), intermolecular forces dominate over any influence of the surface, while the same molecules adsorbed onto the (110) face feel a much stronger surface impact leading to horizontal adsorption of molecules. Nevertheless, a favorable Sn-down position for molecules on rutile (110) remains a thought-provoking phenomenon. The XPS results described above suggest that the switching of the molecules is not accompanied by the creation of new chemical bonds. The interaction likely originates from a physical background. The characteristic feature of saucer-shaped phthalocyanines, such as SnPc, is the presence of a dipole moment. The tin atom is positively charged, and hence, a dipole moment perpendicular to the macrocycle is directed towards the metal atom. However, the picture of a simple dipole is misleading since the charge is distributed either under or above the macrocycle plane of such a nonplanar Pc molecule. As a result, the abovementioned relaxation of a Sn-down Pc molecule leads to an increase in the dipole moment from −0.6 D for gas-phase SnPc to −1.13 D for relaxed SnPc. This effect is mainly due to a change of the position of positively charged H atoms at the periphery of the molecule towards the surface.

Also, the electrostatic landscape of a reduced rutile (110) surface prepared in UHV by sputter–anneal cycles is complicated. The cleaning procedure creates oxygen vacancies, which in turn leads to the formation of polarons near the surface [29–31]. Additionally, water molecules may dissociate at oxygen vacancies and form hydroxy groups on the surface. Both species can be recognized in our STM images and they both lead to the formation of dipole moments pointing away from the surface [32]. Their local arrangement may play a significant role in the interaction between a molecular adsorbate and the substrate, as was presented for CuTPP on rutile (110) [33]. The charge state of a CuTPP molecule depends on the particular localization of hydroxy groups under the molecule. For Sn-down Pc molecules, the relaxation brings the macrocycle closer to the surface, which increases long-range dispersive forces. The fact that the Sn-down geometry is preferred could be rationalized by assuming that the Sn atom locates between surface hydroxy groups, allowing for attractive interactions between positive surface defects and negatively charged pyrrole-like subunits. Such a geometry can minimize the interaction energy (van der Waals and electrostatic) even at the expense of molecule deformation.

Another issue that needs to be addressed is the rotation of the manipulated molecules. The majority of Sn-down molecules adsorb over titanium rows with their diagonal parallel to the [001] direction of the rutile (110) surface. Tip-induced switching sometimes leads to the rotation of the molecule with respect to the surface directions. We suggest that this is the result of the abrupt nature of tip-induced manipulation in which a molecule does not have enough time to thermalize and adapt the optimal position. In the case of SnPc, the interaction is mainly concentrated in the central area of the molecule. The orientation of the molecule on the surface reflects the accommodation to the corrugated morphology of the substrate and is directed by weak physical forces. Consequently, the energy gain through the correct orientation is quite small. This is in contrast to molecules possessing anchoring moieties at the edges for which the rotation can lead to significant changes. Such a phenomenon was observed for CuTPP deposited onto the TiO_2_(110) surface [34], where molecular rotation allowed for the creation of additional bonds between the carboxylic groups and the surface.

## Conclusion

The adsorption process of SnPc molecules on the (110) face of rutile TiO_2_ was studied by microscopic techniques supported by XPS analysis and DFT simulations. At room temperature, single molecules adsorb onto the TiO_2_ surface in a flat-lying position, but their mobility renders imaging impossible. With increasing coverage, increasingly more molecules appear to be stable due to either the influence of surface defects or steric interactions. However, SnPcs do not form a well-defined, regular structure, which suggests that interactions between adjacent flat-lying molecules are negligible unless they partly overlap. After deposition, saucer-shaped SnPcs may adsorb onto the rutile (110) surface with the tin atoms pointing either towards (Sn-down) or away (Sn-up) from the surface, as shown by LT-STM measurements combined with DFT-based simulations. Sn-up molecules switch irreversibly to the down position as a result of sample annealing above 200 °C, as well as a result of tip-induced manipulation, while adsorbed Sn-down molecules remain intact. The annealing to temperatures above 280 °C leads to the desorption of the molecules. The change of the molecular positions is not accompanied by the creation of new chemical bonds, as XPS spectra reveal. This suggests that positioning the Sn atom in close proximity to the surface does not induce a significant charge transfer between the substrate and the molecule. Due to the non-flat shape of the molecules and their ability to bend the pyrrole-like subunits, the Sn-down position better adjusts to the corrugated rutile (110) surface, while the orientation of an electrical dipole moment towards the substrate most likely acts as an additional stabilizing factor.

## Supporting Information

File 1 Additional experimental data.
