# Supplementary material for: Adsorption behavior of tin phthalocyanine onto the (110) face of rutile TiO2
Source: Beilstein J Nanotechnol. 2020 May 26;11:821–8. doi: 10.3762/bjnano.11.67 (PMC7277932; doi:10.3762/bjnano.11.67)
Supplement: File 1 — Additional experimental data. [file Beilstein_J_Nanotechnol-11-821-s001.pdf]

BEILSTEIN JOURNAL OF NANOTECHNOLOGY

Supporting Information

for

Adsorption behavior of tin phthalocyanine onto the (110) face of rutile TiO_2

Lukasz Bodek, Mads Engelund, Aleksandra Cebrat and Bartosz Such

Beilstein J. Nanotechnol. **2020,** *11,* 821–828. doi:10.3762/bjnano.11.67

Additional experimental data

License and Terms: This is a supporting information file under the terms of the Creative Commons Attribution License (http://creativecommons.org/licenses/by/4.0). Please note that the reuse, redistribution and reproduction in particular requires that the authors and source are credited.
The license is subject to the *Beilstein Journal of Nanotechnology* terms and conditions: (https://www.beilstein-journals.org/bjnano)

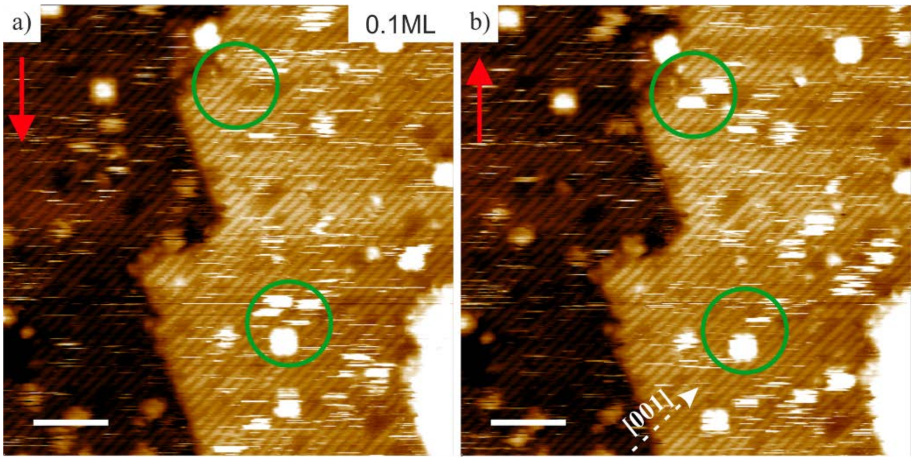

Figure S1: (a, b) Empty-state STM images of SnPc molecules on the rutile TiO_2(110) surface at low coverage (approx. 0.1 ML) taken at room temperature. Panels a) and b) present the same scanned area with a red arrow indicating the slow scan direction. Green circles show regions of tip-induced molecular movement. Streaky horizontal lines correspond to mobile molecules. Some of molecules remain stable under the scanning conditions. Scanning parameters: 30 × 30 nm^2; I_t = 1.2 pA, U_{tip} = 0.81 V. Scale bars: 5 nm.

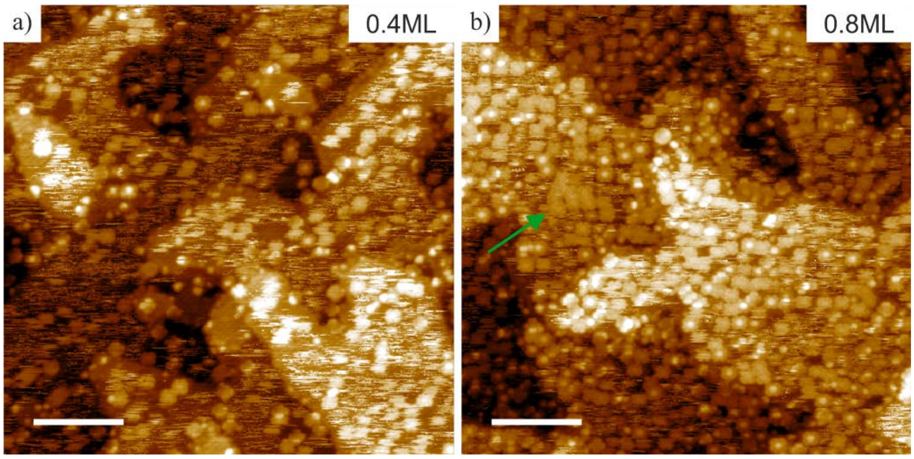

Figure S2: Empty-state STM image of the rutile (110) surface after deposition of (a) 0.4 ML SnPc and (b) 0.8 ML SnPc. a) A small fraction of molecules appears stable during scanning, while streaky areas indicate the presence of many mobile molecules; b) the majority of adsorbed molecules remains stable. The green arrow indicates a region of several arranged molecules between randomly distributed molecules. Scanning parameters: 50 × 50 nm^2; a) I_t = 2 pA, U_{tip} = 1 V; b) I_t = 1.7 pA, U_{tip} = 1 V; room temperature. Scale bars: 5 nm.

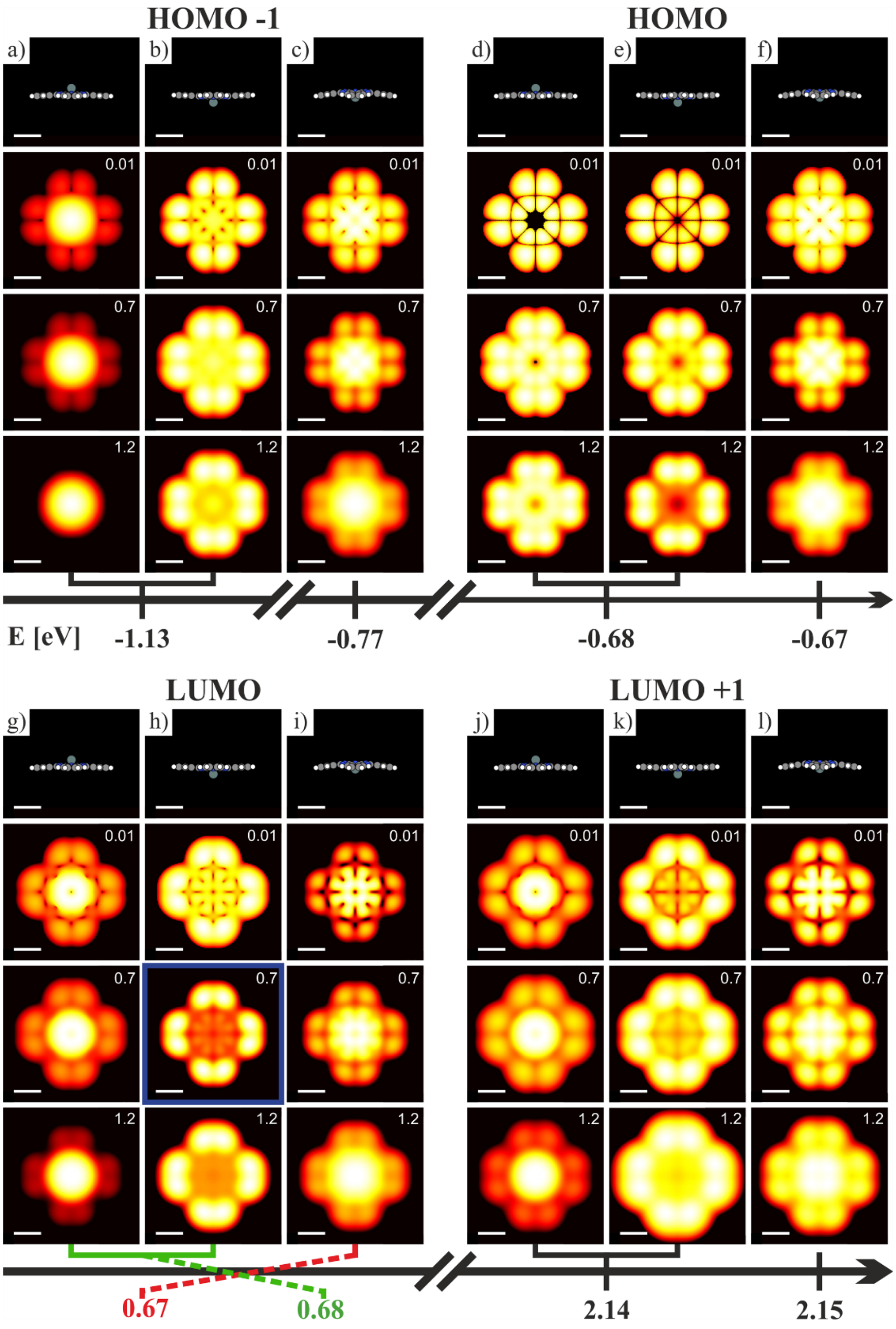

Figure S3: DTF-optimized geometry of a) gas-phase SnPc-up, b) gas-phase SnPc-down and c) SnPc-down influenced by a corrugated rutile (110) surface with corresponding simulated STM images at different values of spatial broadening (0.01 Å; 0.7 Å; 1.2 Å) at the HOMO−1 energy level. Sets of simulated STM images for each geometry are presented at (d–f) HOMO, (g–i) LUMO and (j–l) LUMO+1 energy levels. The calculated position of the orbitals (energy value) for each case is given below the corresponding column. The energy scale is the electron eigenvalues, the same scale probed by the bias voltage. Scale bars: 0.5 nm.

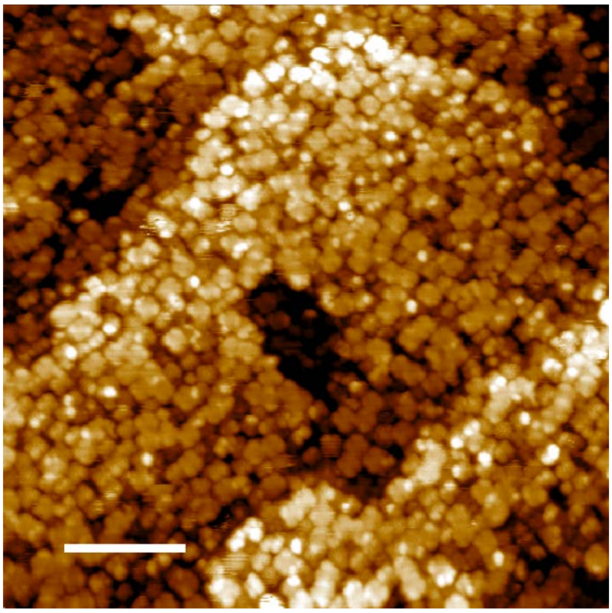

Figure S4: Empty-state STM image of a rutile (110) surface after the deposition of three monolayers equivalent, annealed at 200 °C to remove mobile admolecules. Molecules adsorb with their macrocycle parallel to the surface without well-defined arrangement. Scanning parameters: 50 × 50 nm^2; a) I_t = 2.3 pA, U_{tip} = 1 V; room temperature. Scale bar: 5 nm.

Calculation methodology for Figure 3 and Figure S3

The heuristic model introduced to account for the surface interaction consists of two components: (1) a weak potential designed to mimic a weak surface interaction and (2) the fixture of the height of the Sn atom to model the strong interaction between the atom and the surface. The results depend weakly on the strength of the model potential,

$$E(z) = 0.2x\left(\frac{1}{(z-2)^2} - \frac{1}{z-2}\right) \text{ [eV]},$$

while the Sn height simply changes the relative prominence of the center and the edges in the STM image. More consistency was obtained after fixing the Sn height to −0.4 Å resulting in the DFT simulation images presented in Figure 3 and Figure S3.
